# Social Isolation Among Older Adults With Dysphagia Requiring Home Health Care: A Cross-Sectional Study

**DOI:** 10.7759/cureus.77528

**Published:** 2025-01-16

**Authors:** Reiko Yoshizaki, Ayako Nakane, Kanako Yoshimi, Takayuki Saito, Mitsuko Saito, Kohei Yamaguchi, Kazuharu Nakagawa, Jun Aida, Haruka Tohara

**Affiliations:** 1 Department of Dysphagia Rehabilitation, Graduate School of Medical and Dental Sciences, Institute of Science Tokyo, Tokyo, JPN; 2 Department of Dentistry and Oral Surgery, Tokyo Shinjuku Medical Center, Tokyo, JPN; 3 Department of Dentistry, Gohan ga Tabetai Dental Clinic, Tokyo, JPN; 4 Department of Dental Public Health, Graduate School of Medical and Dental Sciences, Institute of Science Tokyo, Tokyo, JPN

**Keywords:** aged, dysphagia, home care patient, interaction status, social isolation

## Abstract

Background and objectives: We hypothesized that community-dwelling older adults with impaired swallowing functions have a higher risk of social isolation than individuals without impaired function. The aim of this study was to identify the association of dysphagia with social isolation in home care patients using daily interaction frequency and laughter frequency as indicators of social interaction.

Methods: One hundred ninety home care patients, aged ≥65 years, were included in this cross-sectional questionnaire survey. The patients’ basic information, level of care required, and social background as a percentage of public health care costs were obtained. Swallowing function, assessed with the Food Intake Level Scale (FILS), was evaluated from the medical records. Respondents answered how often they interacted with people outside their family, the number of people they interacted with, and how often they laughed in their daily lives. Logistic regression analyses were performed to examine factors associated with social isolation.

Results: Laughter frequency was significantly lower in the dysphagia group (P=0.011). The interaction frequency was lower in those who needed more care (odds ratio (OR) (95% confidence interval (CI)) = 0.16 (0.03, 0.78)) and lower in those with dysphagia (0.40 (0.17, 0.97)). The number of exchanges was lower for those with dysphagia (0.22 (0.09, 0.58)) and needed more care (0.10 (0.02, 0.60)) and higher for those with dementia (2.86 (1.22, 6.66)).

Conclusions: Swallowing function is significantly associated with social interaction between homebound older adults and others. An approach to maintaining and improving swallowing function in older people requiring home healthcare may help maintain social interaction and prevent social isolation.

## Introduction

With a rapidly aging population globally, addressing social isolation and loneliness among older adults is critical. Social isolation is defined in terms of people's social network structure and reflects an objective lack of social relationships. Loneliness is a subjective phenomenon that reflects the perception of a lack of emotional intimacy and social needs [1].

Factors contributing to social isolation and loneliness among older adults include personal factors, the availability of social support, environmental factors, poverty, and disease [2,3]. With regard to personal factors, social isolation decreases cognitive function [2] and health behaviors. It also decreases the availability of health support, which then increases the incidence of cerebrovascular disease, increases the rates of rehospitalization after cerebrovascular disease, and decreases survival rates [4]. Social isolation is associated with mortality [3], as are cardiovascular disease and mental health [4]. Poverty, smoking, alcohol consumption, obesity, and frailty are risk factors for increased mortality. Individuals who are socially isolated have a 50% higher mortality rate than individuals who are not [5].

Older adults are prone to oral and swallowing dysfunctions because of aging, frailty, sarcopenia, and disease [6]. Significantly frailer older adults have impaired swallowing function [7]. Social frailty can be a risk factor for future physical frailty and is significantly associated with overall muscle weakness [8]. In a study of independent community-living older adults, psychological factors such as depression significantly affected the frequency of outings because of oral function decline. Furthermore, in a survey study of older patients requiring long-term care who were cared for at home, it was reported that eating and swallowing functions were significantly maintained in individuals who enjoyed their daily lives with a high quality of life [9]. Thus, the decline in oral and swallowing function in older adults is not necessarily limited to functional impairment but may also be related to social activities and quality of life. However, whether the presence or absence of dysphagia in older adults is directly associated with social isolation is unclear. While previous studies have investigated factors such as cognitive function, frailty, and depression in relation to social isolation, few studies have specifically examined the role of swallowing dysfunction. This study addresses this gap by investigating the association of dysphagia with social interaction and social isolation in home care patients.

Based on the evidence that oral and swallowing dysfunctions can affect the quality of life and social engagement, we hypothesized that community-dwelling older adults with impaired feeding and swallowing functions would have a greater risk of social isolation than older adults without these problems. Therefore, this study focused on dysphagia in older adults and aimed to clarify the association between social interaction status and swallowing function in home care patients, using social interaction status and laughter frequency as indicators of social isolation [10].

## Materials and methods

Participants

This cross-sectional study used a questionnaire-based survey conducted with 190 patients receiving dental care visits from Tokyo Medical and Dental University Hospital (Institute of Science Tokyo) or Gohan ga Tabetai Dental Clinic or Sunflower Building Dental Clinic in Tokyo, Japan, between August 2021 and July 2023. Surveys were hand-delivered, mailed, or web-responded. Eligibility criteria were men and women aged ≥65 in home care and those who agreed to participate and complete the questionnaire. Exclusion criteria were institutionalized or hospitalized patients and patients with Japan Coma Scale scores of 100-300. Proxy answered questions for patients with dementia. Special care was taken to minimize any burden on elderly participants during the survey process.

The study was approved by the Dental Research Ethics Committee of Tokyo Medical and Dental University (Tokyo, Japan; approval ID: no. D2020-087). Written informed consent was obtained from all participants or their legal representatives, with the consent form verbally explained to ensure understanding. This study was a human observational study. The manuscript conforms to the Strengthening the Reporting of Observational Studies in Epidemiology (STROBE) guidelines. The survey adhered to ethical guidelines based on the Declaration of Helsinki.

Data collection

By using patients’ medical records, data on age, sex, household composition, primary illness, dementia status, care needs level, and medical and nursing care services were collected. The level of care needed is an indicator of Japan’s official social security system, representing the extent of daily living assistance required. It is classified into eight levels: (1) care needs level 5; (2) care needs level 4; (3) care needs level 3; (4) care needs level 2; (5) care needs level 1; (6) support needs level 2; (7) support needs level 1; and (8) not applicable. For the purpose of this study, we assigned numerical values to each level and calculated the median accordingly. The values displayed in Table 1 correspond to these assigned levels, where 1 represents care needs level 1, 2 represents care needs level 2, 3 represents care needs level 3, and so on up to 5, which represents care needs level 5.

Support need refers to a condition in which a person can manage daily life independently but requires partial assistance. Care need refers to a condition necessitating assistance in all aspects of daily living. Details regarding specific levels of support needs and care needs are provided in Appendix A. 

Social isolation is associated with low income and poverty [11]. Therefore, this study examined the percentage of copayments for public medical insurance as an indicator of participants’ living conditions. In Japan, insurance coverage for medical expenses varies by age and income status. Individuals aged ≤69 years pay 30%, those aged 70-74 years pay 20%, and those aged ≥75 years pay 10%. However, these percentages can change based on income level, with higher-income individuals aged ≥75 paying 20% or 30%. Financially distressed individuals do not have to make copayments.

Swallowing function was assessed using the Food Intake Level Scale (FILS) [12] by extracting information from medical records regarding the participants’ oral intake and food form. Participants were classified into two groups: dysphagia (FILS 1-9) and without dysphagia (FILS 10).

Items related to social isolation

The survey instrument was originally designed for this study, as no similar studies have been conducted previously. Indicators of social isolation were “social interaction status” and “laughter frequency,” referencing previous studies [10,13]. The questionnaire was designed to ensure clarity and ease of understanding for older participants. To minimize the risk of misunderstanding, the survey questions were phrased in simple language, and multiple-choice response options were provided. Additionally, trained interviewers conducted the survey in cases where participants required assistance, ensuring that responses were accurately recorded. While previous studies informed the design of specific questions, this survey was developed specifically for the present study, given its unique focus on dysphagia and social interaction. The detailed contents of the questionnaire are provided in Appendix B.

Interaction status

The frequency and number of interactions were defined as contacts with people other than those living with participants. Respondents answered on a six-point scale for interaction frequency and a five-point scale for the number of people interacted with [13]. Interaction frequency was measured on a six-point scale, where a score of one represents no interaction, and a score of six represents frequent interactions occurring four or more times per week. Higher scores indicate more frequent social interactions. The number of people with social interaction was assessed on a five-point scale, where a score of one indicates no interaction with anyone, and a score of five indicates interaction with 10 or more people. Higher scores reflect interaction with a greater number of people. The specific questions used in the survey, including options for responses, are provided in Appendix B.

Laughter

Laughter was defined as spontaneous (non-artificial) laughter expressions of enjoyment, pleasure, and satisfaction with daily life. Respondents answered, “How often do you laugh in your daily life?” on a four-point scale: “Almost every day,” “One to five times a week,” “One to three times a month,” and “almost never” [10,13].

Data analysis

The sample size was set at 150 individuals, calculated with an effect size of 0.5, an α error of 0.05, a power of 0.8, and an allocation ratio of two (G*Power 3.1; Heinrich-Heine-Universität Düsseldorf, Düsseldorf, Germany).

Interaction frequency was divided into two groups: more than four times weekly and less than four times weekly. The number of people interacted with was divided into two groups: “interacting with ≥10 people” and “interacting with <10 people” [13]. Laughter frequency was grouped into: “laughing nearly every day” or “laughing less than five times weekly” [10].

The Wilcoxon rank-sum test was used for each item to compare the dysphagia group with the group without dysphagia. Multivariable logistic regression analysis was conducted to identify factors associated with social isolation. Interaction frequency, number of people interacting, and frequency of laughter frequency were the objective variables. Age, sex, care needs level, insurance burden ratio, medical conditions, and dysphagia were the explanatory variables. Statistical analyses were performed using IBM SPSS Statistics software, version 28.0.1.1 (IBM Japan, Tokyo, Japan). The significance level was set at 5%.

## Results

Characteristics of the participants

All questionnaires were administered to 190 eligible participants, exceeding the initially calculated sample size of 150. Including additional participants increased the statistical validity. No data were missing, and all data were analyzed. Table 1 shows the participants’ characteristics.

**Table 1:** Patients’ characteristics (N=190) ^1^ Based on the Wilcoxon rank–sum test; *P<0.05. SD: standard deviation; FILS: Food Intake Level Scale Nursing care level: indicates includes eight levels (support needs level 1 to care needs level 5). Levels 1–5 correspond to care needs levels 1–5; Interaction frequency: scored on a scale of one (lowest) to six (highest), where higher scores indicate more frequent social interactions; Number of people with social interaction: scored on a scale of one (lowest) to five (highest), where higher scores indicate interaction with more people.

Patient characteristics	Dysphagia	Without dysphagia	Total
	(n=140)	(n=50)	(n=190)
Sex			
Male n (%)	57 (41)	13 (26)	70 (37)
Female n (%)	83 (59)	37 (74)	120 (63)
Age, years (Mean ±SD)	83.5±8.1	84.9±8.9	83.9±8.3
Nursing care level* (Median, Min.-Max.)	4 (3–5)	2 (1–3)	4 (2–5)
Interaction frequency (Median, Min.-Max.)	6 (4–6)	6 (5–6)	6 (5–6)
Number of people with social interaction* (Median, Min.-Max.)	3 (3–5)	5 (3–5)	4 (3–5)
Laughter frequency* (Median, Min.-Max.)	3 (2–4)	3.5 (3–4)	3 (3–4)
Insurance contribution ratio			
Public assistance	5(4)	6 (12)	11 (6)
10% n (%)	104 (74)	33 (66)	137 (72)
20% n (%)	15 (11)	9 (18)	24 (13)
30% n (%)	15 (11)	2 (4)	17 (9)
Disease			
Cerebrovascular disease n (%)	56 (40)	15 (30)	71 (37)
Dementia n (%)	47 (34)	13 (26)	60 (32)
Neuromuscular disease n (%)	27 (19)	3 (6)	30 (16)
FILS			
Alternative nutrition: FILS 1,2,3 n (%)	31(22)	-	31(16)
Oral intake and alternative nutrition: FILS 4,5,6 n (%)	24(17)	-	24(13)
Oral intake alone: FILS 7,8,9 n (%)	85(61)	-	85(45)
Normal: FILS 10 n (%)	-	50(100)	50(26)

Seventy men were included (average age, 84 years). Of the 190 participants, 20 lived alone, while 170 lived with family members. 180 participants received home visits from a doctor, 137 from nurses, 118 from rehabilitation professionals, and 95 from caregivers (Table 2). Ninety-seven participants utilized day attendance services. The most common insurance contribution rate was 10%, accounting for 72% of participants. The leading diseases were cerebrovascular disease, dementia, and neuromuscular disease. 

**Table 2:** Utilization of home visit services among participants

Type of home visit service n (%)	Dysphagia	Without dysphagia	Total
	(n=140)	(n=50)	(n=190)
Doctor visits	132 (94)	48 (96)	180 (95)
Nurses visits	108 (77)	29 (58)	137 (72)
Rehabilitation professionals visits	92 (66)	26 (52)	118 (62)
Caregivers visits	66 (47)	29 (58)	95 (50)
Day attendance services	69 (49)	28 (56)	97 (51)

With regard to swallowing function, 140 (74%) patients were in the dysphagia group and 50 (26%) patients were in the without dysphagia group. Within the dysphagia group, 85 (46%) required dietary adjustments or restrictions, 31 (16%) had no oral intake, and 24 (12%) received both oral intake and alternative nutrition (Table 1).

Most patients (95%) required some assistance, with 73% requiring significant assistance. Whereas only 3% (6 patients) required support. The dysphagia group had significantly higher care needs levels (P<0.001).

Interaction status and laughter

Fifty-nine percent of participants interacted four or more times weekly, with 37% interacting with at least 10 people and 29% interacting with three to five people. Eleven respondents reported no interactions. Common interactions in the without dysphagia group were with doctors/dentists (96%), nurses (58%), and friends (36%). In the dysphagia group, interactions were mostly with doctors/dentists (94%), nurses (77%), and rehabilitation workers (66%) (Table 3). The number of people interacted with was significantly lower in the dysphagia group (P=0.001). 

**Table 3:** Interaction types in groups with dysphagia and without dysphagia

Type of interaction (%)	Dysphagia	Without dysphagia	Total
	(n=140)	(n=50)	(n=190)
Doctors/Dentists	132 (94)	48 (96)	180 (95)
Nurses	108 (77)	29 (58)	137 (72)
Friends	20 (14)	18 (36)	38 (20)
Rehabilitation workers	92 (66)	26 (52)	118 (62)

The laughter frequency was nearly every day in 41% (n=78) and one to five times per week in 39% (n=74) of respondents, accounting for 80% of the total. Thirteen (7%) respondents reported laughing approximately one to three times per month, and 25 (13%) respondents did not laugh. The laughter frequency was significantly lower in the dysphagia group (P=0.011).

Factors influencing interaction frequency, number of people interacting, and laughter frequency

The results of univariate and multivariate logistic regression analyses are in Tables 4-6. Multivariate analysis showed that those with higher levels of nursing care needs (odds ratio (OR) (95% confidence interval (CI)) = 0.16 (0.03, 0.78)) and dysphagia (0.40 (0.17, 0.97)) were likely to interact less frequently (Table 4). Regarding the number of people interacting, those with dysphagia (0.22 (0.09, 0.58)) and levels of nursing care needs (0.10 (0.02, 0.60)) were likely to interact with fewer people. Those with dementia (2.86 (1.22, 6.66)) were likely to have more interactions (Table 5). No factors significantly influenced laughter frequency (Table 6).

**Table 4:** Association of dysphagia and related items with interaction frequency by logistic regression analysis *P<0.05; OR: odds ratio; CI: confidence interval; FILS: Food Intake Level Scale

		Interaction frequency	Univariable			Multivariable		
		4 times ≥week, n(%)	OR	95% CI	P-value	OR	95% CI	P-value
Sex								
Male		44 (63)	1.00			1.00		
Female		68 (57)	1.29	(0.71, 2.37)	0.40	1.37	(0.69, 2.72)	0.37
Age, years		112 (59)	1.01	(0.97, 1.05)	0.61	0.99	(0.95, 1.04)	0.80
Nursing care level								
None, Support needs levels 1, 2		4 (40)	1.00			1.00		
Care needs level 1		11 (50)	0.67	(0.15, 3.04)	0.60	0.45	(0.09, 2.29)	0.33
Care needs level 2		9 (45)	0.82	(0.17, 3.81)	0.80	0.55	(0.11, 2.87)	0.48
Care needs level 3		20 (59)	0.47	(0.11, 1.97)	0.30	0.26	(0.05, 1.30)	0.10
Care needs level 4		24 (67)	0.33	(0.08, 1.41)	0.14	0.16	(0.03, 0.84)	0.03*
Care needs level 5		44 (65)	0.36	(0.09, 1.42)	0.15	0.16	(0.03. 0.78)	0.02*
Insurance contribution ratio								
Public assistance		8 (73)	1.00			1.00		
10%		75 (54)	2.24	(0.57, 8.80)	0.25	1.76	(0.38, 8.23)	0.47
20%		18 (75)	0.89	(0.18, 4.48)	0.89	0.77	(0.13, 4.45)	0.77
30%		11 (65)	1.46	(0.28, 7.64)	0.66	1.05	(0.17, 6.30)	0.96
Disease								
Cerebrovascular disease	Absent	70 (59)	1.00			1.00		
	Present	42 (59)	0.99	(0.54, 1.79)	0.96	1.34	(0.68, 2.65)	0.39
Dementia	Absent	77 (59)	1.00			1.00		
	Present	35 (58)	1.04	(0.56, 1.93)	0.91	1.07	(0.52, 2.20)	0.85
Neuromuscular disease	Absent	98 (61)	1.00			1.00		
	Present	14(47)	1.81	(0.82, 3.96)	0.14	2.06	(0.83, 5.11)	0.12
Dysphagia								
FILS level 10		33 (66)	1.00			1.00		
FILS < level 10		79 (56)	0.67	(0.34, 1.31)	0.24	0.40	(0.17, 0.97)	0.04*

**Table 5:** Association of dysphagia and related items with number of people with social interactions by logistic regression analysis *P<0.05; **P<0.01; OR: odds ratio; CI: confidence interval; FILS: Food Intake Level Scale

		Interacting with ≥10 people, n(%)	Univariable			Multivariable		
			OR	95% CI	P-value	OR	95% CI	P-value
Sex								
Male		30 (43)	1.00			1.00		
Female		40 (33)	1.50	(0.82, 2.75)	0.19	1.76	(0.84, 3.71)	0.14
Age, years		70 (37)	1.00	(0.96, 1.03)	0.93	0.95	(0.90, 0.99)	0.03*
Nursing care level								
None, Support needs levels 1, 2		3 (30)	1.00			1.00		
Care needs level 1		9 (41)	0.62	(0.13, 3.06)	0.56	0.29	(0.05, 1.78)	0.18
Care needs level 2		3 (15)	2.43	(0.39, 15.1)	0.34	1.11	(0.15, 8.07)	0.92
Care needs level 3		16 (47)	0.48	(0.11, 2.18)	0.34	0.16	(0.03, 0.93)	0.04*
Care needs level 4		9 (25)	1.29	(0.27, 6.05)	0.75	0.33	(0.05, 2.09)	0.24
Care needs level 5		30 (44)	0.54	(0.13, 2.28)	0.40	0.10	(0.02, 0.60)	0.01*
Insurance contribution ratio								
Public assistance		7 (64)	1.00			1.00		
10%		44 (32)	3.74	(1.04, 13.4)	0.04*	3.38	(0.71, 16.0)	0.13
20%		9 (38)	2.92	(0.66, 12.8)	0.16	3.49	(0.61, 19.9)	0.16
30%		10 (59)	1.23	(0.26, 5.84)	0.80	0.87	(0.15, 5.27)	0.88
Disease								
Cerebrovascular disease	Absent	42 (35)	1.00			1.00		
	Present	28 (39)	0.84	(0.46, 1.54)	0.57	1.21	(0.58, 2.50)	0.62
Dementia	Absent	55 (42)	1.00			1.00		
	Present	15 (25)	2.20	(1.11, 4.34)	0.02*	2.86	(1.22, 6.66)	0.02*
Neuromuscular disease	Absent	61 (38)	1.00			1.00		
	Present	9 (30)	1.44	(0.62, 3.34)	0.40	1.45	(0.53, 4.01)	0.47
Dysphagia								
FILS level 10		26 (52)	1.00			1.00		
FILS < level 10		44 (31)	0.42	(0.22, 0.82)	0.01*	0.22	(0.09, 0.58)	0.002**

**Table 6:** Association of dysphagia and related items with laughter frequency by logistic regression analysis OR: odds ratio; CI: confidence interval; FILS: Food Intake Level Scale

		Laughing nearly every day, n(%)	Univariable			Multivariable		
			OR	95% CI	P-value	OR	95% CI	P-value
Sex								
Male		26 (37)	1.00			1.00		
Female		52 (43)	0.77	(0.42, 1.41)	0.40	0.63	(0.31, 1.27)	0.20
Age, years		78 (41)	1.01	(0.97, 1.04)	0.71	1.01	(0.97, 1.06)	0.61
Nursing care level								
None, Support needs levels 1, 2		6 (60)	1.00			1.00		
Care needs level 1		15 (68)	0.70	(0.15, 3.30)	0.65	0.53	(0.10, 2.70)	0.44
Care needs level 2		7 (35)	2.79	(0.58, 13.3)	0.20	2.35	(0.47, 11.9)	0.30
Care needs level 3		9 (26)	4.17	(0.95, 18.2)	0.06	3.70	(0.77, 17.8)	0.10
Care needs level 4		15 (42)	2.10	(0.50, 8.76)	0.31	1.86	(0.38, 9.04)	0.44
Care needs level 5		26 (38)	2.42	(0.62, 9.41)	0.20	1.95	(0.42, 8.99)	0.40
Insurance contribution ratio								
Public assistance		6 (55)	1.00			1.00		
10%		54 (39)	1.87	(0.54, 6.42)	0.32	1.64	(0.39, 6.98)	0.50
20%		14 (58)	0.86	(0.20, 3.61)	0.83	0.73	(0.15, 3.50)	0.69
30%		4 (24)	3.90	(0.76, 20.0)	0.10	3.38	(0.55, 20.7)	0.19
Disease								
Cerebrovascular disease	Absent	48 (40)	1.00			1.00		
	Present	30 (42)	0.92	(0.51, 1.68)	0.80	0.90	(0.45, 1.79)	0.76
Dementia	Absent	57 (44)	1.00			1.00		
	Present	21 (35)	1.45	(0.77, 2.73)	0.25	1.30	(0.62, 2.76)	0.49
Neuromuscular disease	Absent	69 (43)	1.00			1.00		
	Present	9 (30)	1.77	(0.76, 4.10)	0.18	1.83	(0.69,4.87)	0.22
Dysphagia								
FILS level 10		25 (50)	1.00			1.00		
FILS < level 10		53 (38)	0.61	(0.32, 1.17)	0.14	0.89	(0.38, 2.09)	0.80

## Discussion

This study aimed to identify factors associated with social isolation in home care patients using daily interaction status and laughter frequency as indicators. Dysphagia and care needs levels were associated with the frequency and number of people respondents interacted with others. Individuals requiring more care and those with lower swallowing function had fewer interactions. Individuals with dementia had more social interactions. Age, sex, care needs, insurance burden ratio, presence of primary illness, and swallowing function were not associated with laughter frequency.

Characteristics of the interaction status of home care patients

Most participants required nursing care in all aspects of daily life, and their interaction status was mostly limited to medical personnel. People with physical disabilities are more likely to be isolated [14], and homebound older adults who were independent in walking, bathing, and toileting reported that an interaction frequency of less than once weekly led to deteriorating health status, and less than once monthly led to early death [13]. The number of interactions in the dysphagia group was lower than that in the without dysphagia group; however, the interaction frequency was at least four times weekly. All participants in this study received home visits from a physician or dentist. Most participants received home services from a nurse or caregiver. The participants’ interactions with society were likely maintained via their interactions with medical personnel. The continuity of regular services, weekly or multiple times per month, probably did not lead to the severe isolation reported in previous studies [13].

Many participants in this study lived with family members, such as spouses or children. In Japan, the number of family caregivers is decreasing due to the falling birth rate, the aging population, and the trend toward nuclear families. This change increases the caregiving burden [15]. 

According to a 2022 survey by the Ministry of Health, Labor, and Welfare [16], over half of primary caregivers living with care recipients were over 65 years old. The burden of older caregivers is significant, particularly as traditional family structures weaken [17].　Therefore, interactions may be limited to the minimum necessary for caregiving, affecting the interaction frequency. However, increased use of home and other services has decreased the level of care required [18]. Therefore, using home services, revitalizing local communities, and enhancing financial support may help maintain social interactions and physical function.

Relationship between social isolation and swallowing function

For older adults, food is important not only for nutrition but also as a social activity, affecting their quality of life [19]. Although excluding dysphagia, one of the isolation factors may help prevent isolation, the dysphagia group in this study required more care than those without dysphagia. Approximately 30% of the patients were on tube feeding and without oral intake. These patients often face mobility restrictions and increased assistance needs, which can reduce their opportunities to interact socially. In one study [20] investigating disease-specific quality of life in patients with dysphagia, the presence of dysphagia, oral intake, and differences in food form were closely associated with quality of life. However, unlike the current study, which focused on home care patients, previous studies often included community-dwelling or institutionalized older adults, which may explain the differences in interaction frequency. Home care patients tend to have more limited mobility, reducing their opportunities for spontaneous social interactions. Therefore, early intervention by healthcare professionals is crucial to prevent severe dysphagia.

Some studies also report that food provides an opportunity to interact with others and a way to cope with social isolation [21]. Furthermore, reports indicate improved nutritional status among home care patients when home-delivered meals and appropriate social support interventions are provided [22]. Providing settings where patients with dysphagia can enjoy eating out or interact with others through food may help prevent isolation. Public awareness and education about dysphagia could further support these efforts.

Relationship between social isolation and cognitive function

Dementia also influences the number of people interacting. Social isolation is known to negatively affect cognitive function in older adults, potentially leading to cognitive decline and poorer health outcomes [23]. Low social activity adversely affects cognitive and executive function, working memory, visuospatial ability, and processing speed [24]. Conversely, increased physical and social activity can prevent cognitive decline [25]. Our study suggests that the presence of dementia may require older adults to need more support from others in order to live in the community. This result aligns with previous findings [23] but may differ in magnitude due to the study population. Older adults receiving home care typically have more structured interactions through caregiving services, which may buffer against complete social isolation. Therefore, interventions that reduce isolation, enhance social support, and maintain cognitive function may improve older adults’ health and quality of life.

Laughter frequency as an indicator of social isolation in patients with dysphagia

In this section, we discuss laughter frequency as a key indicator of social isolation, focusing on its association with dysphagia to better understand the social engagement of home care patients. Laughter frequency was significantly lower in patients with dysphagia, aligning with findings that swallowing difficulties were strongly associated with stress-reduced enjoyment during meals [26]. Limited laughter is linked to a higher risk of physical disabilities [27]. Although this study did not directly assess subjective dysphagia status, the emotional impact and reduced quality of life associated with dysphagia may contribute to lower laughter frequency.

We expected that more interactions would lead to more opportunities to talk with others and more frequent laughter because the group without dysphagia had more interactions. However, no factors were associated with laughter frequency. Some studies suggest that people laughed 30 times more when in the presence of others than when alone [28]. Thus, the social context might play a more crucial role than the interaction frequency in influencing laughter. Unlike studies conducted in community settings, where social gatherings are more common, the participants in this study predominantly interacted with medical personnel. This difference in interaction context may explain why no factors were strongly associated with laughter frequency despite the observed lower laughter in the dysphagia group. Oral health, including the number of remaining teeth, is also linked to laughter frequency, indicating that maintaining good oral status as well as swallowing function may enhance the quality of life in older adults [29]. Laughter is a form of free communication that requires no words and has no restrictions and could be an important means of communication in patients who may have difficulty with speech. Maintaining social connections through laughter may also improve the physical and mental health of patients with dysphagia.

Limitations

The participants in this study resembled the broader population of patients receiving home dental care in Japan [30]. However, the study only included individuals using public home healthcare, nursing, and care services and did not compare these patients with those not receiving such support. Thus, generalizing the findings requires caution, as the study population may have distinct social characteristics. Additionally, all facilities were in Tokyo, an urban area with a higher population density, which may limit the findings' applicability to rural settings. Self-reported data may introduce recall bias. Future research could expand the comparison to various groups to identify effective measures against social isolation. Lastly, as a cross-sectional study, this research cannot determine the direction of causality between declined swallowing function and decreased social interaction. Longitudinal studies are needed to explore these relationships further.

## Conclusions

Dysphagia was significantly associated with interactions with others among older home care patients. Individuals with high care needs and poor swallowing function had significantly lower frequencies of interactions with others. Although the generalizability of the findings to other populations, such as younger individuals or those with different health conditions, is limited, individuals who require advanced medical care owing to aging or illness and have swallowing disorders are at an increased risk of social isolation. An approach to maintain and improve swallowing function in older home care patients may help maintain social interactions and prevent social isolation.

## Appendices

Appendix A

Support Needs Levels

Support need refers to a condition in which a person can manage daily life independently but requires partial assistance.

Support needs level 1: The individual can perform basic daily activities independently but requires supervision or assistance for certain tasks.

Support needs level 2: The individual experiences muscle weakness, is unstable in walking and standing, and is at a high risk of needing care.

Care Needs Levels

Care need refers to a condition necessitating assistance in all aspects of daily living.

Care needs level 1: The individual requires partial assistance with daily activities, standing up, and walking, and has slight cognitive decline.

Care needs level 2: The individual needs more care for daily activities than that of level 1 and exhibits cognitive decline.

Care needs level 3: The individual requires comprehensive assistance with daily activities; requires a cane, walker, or wheelchair to stand up and walk; has cognitive decline; and requires supervision.

Care needs level 4: The individual needs more assistance in all aspects of daily living than at level 3 and has a significant decline in thinking and understanding abilities.

Care needs level 5: The individual requires assistance with all daily activities and has difficulty communicating.

Appendix B

Questionnaire Survey

1 Please indicate the sex of the patient. Mark only one option. Male

Female

2 Please indicate the patient's age.

3 Please select the relevant category of the care required.

Not applicable

Support needs Level 1

Support needs Level 2

Care needs Level 1

Care needs Level 2

Care needs Level 3

Care needs Level 4

Care needs Level 5

4 How often do you (the patient) personally meet, go out with, or communicate via social networking sites, e-mail, or phone with friends, acquaintances, neighbors, nonresident family members, or relatives (including assistance from caregivers)? (Please answer, based on the situation before COVID-19.) Mark only one option.

4 or more times per week

2-3 times per week

Once per week

1-3 times per month

A few times per year

Not at all

5 How many friends, acquaintances, neighbors, nonresident family members, or relatives have you (the patient) personally met in the past month? Each person is counted only once, regardless of the number of times you met them. (Please answer, based on the situation before COVID-19.) Mark only one option.

0 people (none)

1-2 people

3-5 people

6-9 people

10 or more people

6 What is the relationship between friends or acquaintances that you (the patient) frequently meet? Please check all that apply.

Neighbors or people from the same area

Friends

Colleagues or former colleagues

Doctors or dentists

Visiting nurses

Helpers

Rehabilitation professionals

Day service staff

Other (Please specify this in the next section)

7 How often do you (the patient) have opportunities to laugh in your daily life? Mark only one option.

Nearly every day

About 1-5 times per week

About 1-3 times per month

Rarely
